# Prevalence of Atopic Dermatitis Among Children Under 19 in an East-Hungarian Agricultural County

**DOI:** 10.1080/17402520600565415

**Published:** 2006

**Authors:** Agnes Kuhnyar, Katalin Egyud, Imre Szabo, Janos Hunyadi, Lajos Kosa

**Affiliations:** 1 1st Pediatric Institute Pediatrics of Svabhegy Semmelweis University 6. Martonhegyi str Budapest 1121 Hungary; 2 Department of Dermatology Nyiregyhaza Hospital 68. Szent Istvan str Nyíregyháza 4400 Hungary; 3 Department of Dermatology Medical and Health Science Center University of Debrecen 98. Nagyerdei krt Debrecen 4012 Hungary

## Abstract

The prevalence of atopic dermatitis has significantly increased in developed countries during the past several decades. Surveys performed in Hungary also show a growing number of atopic dermatitis (AD) cases, although, a carefully designed case-controlled studies have not been performed. Therefore, we investigated the prevalence of AD in individuals under 19 years of age within the agricultural area of East-Hungary. Combined data obtained with Schultz-Larsen questionnaire on 1158 children were analyzed, and 25% of the index persons were examined by dermatologist. The mean prevalence of AD determined by questionnaires appeared to be 17.5% in the entire study population. Result of dermatological examination verified the validity and sensitivity of the questionnaire. A negative correlation was found between the severity of the disease and the length of breast feeding period. (Spearman's correlation coefficient = − 0.2247, *p* = 0.034). The prevalence of AD in an East-Hungarian agricultural area is nearly as high as that reported for populations residing in industrially developed countries, with a higher prevalence during childhood. Data suggest that premature abruption of breast feeding maybe one of the major factors among other environmental factors that is contributing to the development of AD.


*Clinical & Developmental Immunology*, June–December 2006; 13(2–4): 395–399

Taylor & Francis

Taylor & Francis Group

### Prevalence of atopic dermatitis among children under 19 in an East-Hungarian agricultural county

AGNES KUHNYAR[1], KATALIN EGYUD[2], IMRE SZABO[1], JANOS HUNYADI[3], & LAJOS KOSA[1]

[1]*1st Pediatric Institute, Pediatrics of Svabhegy, Semmelweis University, 6. Martonhegyi str, Budapest 1121, Hungary,* [2]*Department of Dermatology, Nyiregyhaza Hospital, 68. Szent Istvan str, Nyíregyháza 4400, Hungary, and* [3]*Department of Dermatology, Medical and Health Science Center, University of Debrecen, 98. Nagyerdei krt, Debrecen 4012, Hungary*

**Abstract**

The prevalence of atopic dermatitis has significantly increased in developed countries during the past several decades. Surveys performed in Hungary also show a growing number of atopic dermatitis (AD) cases, although, a carefully designed case-controlled studies have not been performed. Therefore, we investigated the prevalence of AD in individuals under 19 years of age within the agricultural area of East-Hungary. Combined data obtained with Schultz-Larsen questionnaire on 1158 children were analyzed, and 25% of the index persons were examined by dermatologist. The mean prevalence of AD determined by questionnaires appeared to be 17.5% in the entire study population. Result of dermatological examination verified the validity and sensitivity of the questionnaire. A negative correlation was found between the severity of the disease and the length of breast feeding period. (Spearman's correlation coefficient $= -0.2247$, $p = 0.034$). The prevalence of AD in an East-Hungarian agricultural area is nearly as high as that reported for populations residing in industrially developed countries, with a higher prevalence during childhood. Data suggest that premature abruption of breast feeding maybe one of the major factors among other environmental factors that is contributing to the development of AD.

**Keywords:** *Atopy, atopic dermatitis, breast feeding, childhood, prevalence*

### Introduction

Atopic dermatitis (AD) (also called atopic eczema) is an inflammatory, chronically relapsing, non-contagious and extremely pruritic skin disease, which frequently starts early in childhood. Atopic dermatitis together with allergic bronchial asthma and allergic rhinoconjunctivitis belongs to the group of "atopic diseases". The genetic basis of this disease has been studied by a number of groups and found to have multifactorial traits with gene loci localized to several chromosomes. A genetic basis of this disease is also supported by the finding of a 75–85% concordance in monozygotic twins and as expected a lower 30% in dizygotic twins.

In a subgroup of patients with AD, IgE-mediated allergic reactions play a pathophysiological role. However, there are many patients in whom non-specific factors, such as irritants or psychosomatic influences appear to be of major importance. Therefore, detailed analysis of patients' history is crucial for the correct diagnosis of AD. Moreover, evaluation of allergic sensitization is necessary in each individual.

Clinical symptoms of AD are characterized by polymorphism. The disease often begins with the clinical sign known as "cradle cap" after the first 3 months of life. The symptoms spread to the face and extend to the sides of arms and legs in toddlers, showing extensive oozing and crusting. Later on the typical preferential pattern with eczematous skin lesions of flexures, neck and hands develops, accompanied by dry skin, both as a subjective impression and measurable transepidermal water loss. Lichenification is the result of scratching and rubbing. New exacerbations often start without obvious cutaneous symptoms, with only increased (sometimes localized) itching, followed later on by erythema, papules and infiltration (Wuthrich 1994).

Correspondence: J. Hunyadi, Chair of Department of Dermatology, Medical and Health Science Center, University of Debrecen, 98. Nagyerdei krt, Debrecen 4012, Hungary. Tel: 35 52 442 204. Fax: 36 52 414 632. E-mail: hunyadi@jaguar.dote.hu

ISSN 1740-2522 print/ISSN 1740-2530 online © 2006 Taylor & Francis
DOI: 10.1080/17402520600565415

The clinical diagnosis may be established by the finding of four of the six criteria characteristic for AD: (1) eczematous skin lesions (age dependent); (2) early onset and typical localization of skin lesions according to age; (3) pruritus; (4) stigmata of atopy; (5) personal or family history of atopy; (6) IgE mediated sensitization.

Sebostatis, xerosis, hyperlinearity of palms and soles, linear grooves of fingertips, Dennie–Morgan fold (atopy fold, doubled infaorbital fold), Hertoghe's sign (hypodense or absence of lateral eyebrows), short distance of scalp hair growth to eyebrows (low hairline), periorbital shadow, white dermographism, delayed blanching after intracutaneous injection of acetylcholine and hypersensitivity to wool fabric are the well known stigmata of AD.

Statistical data indicate that the prevalence of AD is dramatically increasing worldwide (Poysa et al. 1991, Severity scoring of atopic dermatitis 1993, Kay et al. 1994, Aberg et al. 1995, von Mutius et al. 1998, Downs et al. 2001, Harangi et al. 2003). Before 1960, the prevalence of the disease was 2–3%, while later on it increased to 9–10% in West-European countries (Schultz and Hanifin 1992). Industrialization is believed to be one of the most important provoking environmental factors. The estimated prevalence of AD based on reports from East- and West-Europe, USA and Australia has been found to be at 15–24% (Kay et al. 1994, Dotterud et al. 1995, 2001, Schultz Larsen et al. 1996, Sugiura et al. 1998, Williams et al. 1999, McNally et al. 2000, Yura and Shimizu 2001, Tay et al. 2002). In Japan a continuous increase was observed between 1985–1993 with the maximum of 24.1%, which was followed by slight decrease later on (Laughter et al. 2000).

Several studies that report an increase in the prevalence of AD have also been reported in Hungary, although, until now well designed case-controlled studies in various regions of Hungary have not been performed. These thoughts prompted us to conduct a carefully designed case-control study in Szabolcs–Szatmar–Bereg, an agricultural county located in Eastern Hungary, in the population under 19 years of age.

### Subjects and methods

### *Study design and population*

The present survey was designed as a cross-sectional questionnaire study of children ranging in age from 0 to 19 using the questionnaire developed by Schultz Larsen et al. (1996) and further improved by Laughter et al. (2000). This questionnaire is based on the most discriminatory features of the Hanifin-Rajka criteria (Hanifin and Rajka 1980, Svensson et al. 1985, Diepgen et al. 1989). It includes statements or questions, each of which is assigned specific scores. If the responder's score reaches certain limit ($>50$), the probability of atopic dermatitis is considered high.

Children under 19 years of age living in Nyiregyhaza, a relatively large city, and three smaller settlements Fehergyarmat, Tuzser and Ujfeherto were chosen for the study. Addresses of the study participants were obtained from the Central Statistical Office (KSH) of Hungary. The total number of individuals within this same age group in the geographic area being studied is 38,248. The 1158 children which represents 3.03% of the index population (Nyiregyhaza: 3.34%; Fehergyarmat: 2.49%, Tuzser: 2.39%, Ujfeherto: 1.00%), was considered sufficient in number for a valid statistical analysis. It was also stated that the number of examined cases was suitable for proving the prevalence of the disease with 95% accuracy at a 1.5 confidence interval, if the prevalence is higher than 10%. Participation in the study was voluntary, with less than 5% refusing to participate in the study. The questionnaires were administered by medical students, who were previously trained with regards to the list of queries and educated on the most important characteristics of AD. The first period of the study was carried out between January and June 2004. After this period, 25% of the index cases were called for dermatological examination in order to evaluate the sensitivity and specificity of the data obtained from the questionnaires.

The data obtained via the questionnaire included information about the gender and the presence of itchy rashes or infantile eczema involving the following skin areas: elbow, knee folds, wrists, ankles, face, neck, hands, arms, legs and the body. Furthermore, the occurrence of unusually dry skin, irritation of skin from textiles (wool), itching of skin when sweating, seasonal variation in severity, worsening by psychological tension or stress, asthma or hay fever (allergic nasal conditions) were also noted. The questionnaires also contained questions concerning the age of patients when skin problems started, the lasting period of itchy rashes (infantile eczema) and familiar history of allergy (infantile eczema, asthma, hay fever, or other allergic nasal conditions).

For the evaluation of the data obtained via the questionnaire, individual questions were scored and finally summarized as suggested by Laughter et al. (2000). Children with a score $\geq 50$ were considered to have AD. The calculated sensitivity is 94.4%, with specificity of 77.6% (positive predictive value: 85.2%, negative predictive value: 97.4%.). Statistical analysis was carried out using the Fisher-, and the Spearman's correlation-test.

### *Dermatological examination*

The second period of the study was carried out in Nyiregyhaza hospital, where the data in the questionnaire were compared with cutaneous symptoms examined by a dermatologist in order to evaluate the sensitivity and specificity of the questionnaires. Three hundred children (including 150 with a score of < 50, and 150 with score of > 50) were called in for examination into the outpatient ambulatory of Nyiregyhaza hospital—BNGI. The atopic status of children and the severity of AD were assessed using the clinical validation and guidelines for the SCORAD index, consensus report of the European Task Force on Atopic Dermatitis. Severity of dermatitis was scored for all patients by the same dermatologist (KE) using standard clinical criteria (SCORing Atopic Dermatitis; SCORAD) (Severity scoring of atopic dermatitis 1993, Kunz et al. 1997). These criteria include measurement of the size of the affected area (A) and scoring the intensity of dermatitis (B) with five levels of intensity: erythema, edema or papulation, oozing or crusting, excoriation and lichenification (each with score 0–2). The SCORAD index was calculated as $A/5 + (7 \times B)/2$ (Severity scoring of atopic dermatitis 1993, Kunz et al. 1997). In addition to the dermatological examination, the birth date, birth-weight and nutrition habits during infancy (duration of breast feeding, beginning of cow milk and supplementary feeding) were also recorded. The skin was inspected extensively for evidence of clinical symptoms and the severity of dermatitis, as described above. On the basis of these data, the SCORAD index for each patient with the diagnosis of AD was calculated. In these cases, the correlation between designated data (i.e. birth weight, breast feeding period, etc.) and the SCORAD index was also calculated. The Specialty training of the physicians (pediatrician, GP or dermatologist) involved in the treatment of the index children was also recorded.

### Results

The skin status and atopic history of children living in an East-Hungarian agricultural urban and rural areas was evaluated using the Schultz-Larsen questionnaire. Based on the parents' responses, the questionnaires were filled by medical students previously trained for the proper interpretation of each question or statement. A total of 1145 questionnaires were properly completed and evaluated in this study.

*Prevalence of AD calculated from the questionnaires' score*

Children with score > 50 were estimated as cases with AD. Two hundred and three persons (17.5%) were

Table I. Gender distribution of the questionnaires' scores.

| Gender | Score | | Total |
|---|---|---|---|
| | ≥50 | <50 | |
| Boys (%) | 95 (16.4) | 475 (83.6) | 568 (49.6) |
| Girls (%) | 108 (18.2) | 472 (81.8) | 577 (50.4) |
| Total (%) | 203 (17.5) | 947 (82.5) | 1.145 (100) |

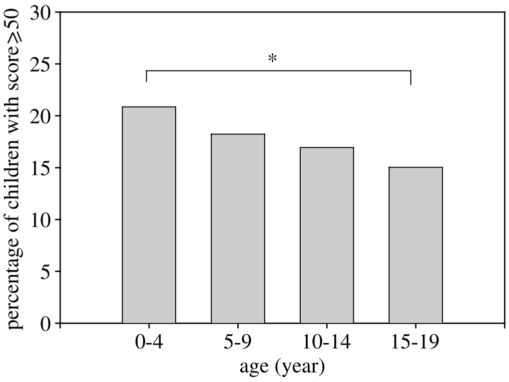

Figure 1. The prevalence of atopic dermatitis in various age groups. The skin status and atopic history of 1145 children living in East-Hungarian agricultural urban and rural areas were evaluated using the Schultz-Larsen questionnaire. Children with score ≥50 were considered to have atopic dermatitis. Comparison of various age groups revealed statistically significant difference between the youngest (age 0–4) and the oldest (year 15–19) children groups with AD. *$p < 0.05$.

found to reach or exceed this score limit. We detected a statistically insignificant trend in female dominance (18.2% female vs. 16.4% male ($p = 0.4143$; Table I). We obtained no statistically significant difference between the prevalence of AD in urban (Nyiregyhaza: 17.4%) as compared with rural (Fehergyarmat, Tuzser, Ujfeherto: 18.25%) areas. The prevalence of AD in children in the age groups of 0–4, 5–9, 10–14 and 15–19 years were 20.86, 18.23, 16.94 and 15.03%, respectively (Figure 1). The difference between the predicted percentage of atopic dermatitis in the age groups of 0–4 and 15–19 years was statistically significant ($p = 0.0493$).

Following analysis of the data from the questionnaires, dermatological examination of representative patients was carried out in Nyiregyháza hospital to confirm the sensitivity and specificity of the Schultz-Larsen questionnaires. Three hundred children (150 with a score of < 50 and 150 with a score of > 50) were initially planned to be examined in the outpatient ambulatory of Nyiregyháza Hospital—BNGI, where the atopic status of children and the severity of the disease was determined using the clinical validation and guidelines for SCORAD index. One hundred and fifty two out of 300 children presented themselves for dermatological examination: 104 with a score of ≥ 50 and 48 with a score of < 50. There was no statistically significant difference ($p = 0.0733$) between the average birth-weight of the two groups of children: 2914.9 ± 495.2 g in group with a score of ≥ 50, and 3.093.8 ± 512.7 g in the group with a score of < 50. We found no correlation between the development of AD and the feeding practices and dietary habits in infancy (Table II). On the other hand, a negative correlation was found between the duration of breast-feeding and the SCORAD value (Table III). Analysis of the data obtained demonstrates that patients with

Table II. Feeding practices and dietary habits in infancy.

| Score | Month | | $p$ |
|---|---|---|---|
| | $\geq 50$ | $< 50$ | |
| Duration of breast feeding | $8.5 \pm 4.7$ | $8.7 \pm 4.0$ | 0.8265 |
| Supplementary feeding | $3.7 \pm 1.7$ | $3.5 \pm 1.2$ | 0.5059 |
| Beginning of cow milk | $11.7 \pm 3.6$ | $12.7 \pm 3.9$ | 0.1967 |
| Mixed feeding | $4.7 \pm 1.4$ | $4.6 \pm 1.1$ | 0.6348 |

a low SCORAD index are generally treated by pediatricians, while dermatologists are taking care of patients with high SCORAD index patients.

Results of the dermatological examination provided the validity and sensitivity of Schultz-Larsen questionnaires. The vast majority of patients who were predicted to have AD based on the high score of the questionnaire were in fact suffering from this disease, while those with score under 50 were not. On the other hand, no correlation between the questionnaire' score and the SCORAD index was detected.

### Discussion

It is well known that the possibility of developing a long lasting AD is higher in those patients who have previously had severe symptoms during early childhood especially in combination with other atopic diseases. Several lines of experimental data prove the existence of a common pathophysiologic link (e.g. elevated serum IgE level, eosinophylia, etc.) between AD, asthma, food allergy and allergic rhinitis. It might also be predicted that the presence of AD increases the risk of asthma development (Iikura et al. 1992, Ninan and Russell 1992).

Statistical data indicate that the prevalence of allergic rhinitis, bronchial asthma and AD is dramatically increasing worldwide (Poysa et al. 1991, Kay et al. 1994, Aberg et al. 1995, von Mutius et al. 1998, Strom et al. 1999, Downs et al. 2001, Harangi et al. 2003). Despite the known social importance of atopic diseases in Hungary, generally there is a low degree of interest in sponsoring a well-organized and comprehensive survey of the prevalence of such diseases. However, there are sporadic publications regarding the prevalence of AD in Hungary. Goncz et al. (1997) carried out screening studies for allergic diseases in 1990 in a group of children age 14–18 and found a 6.1% prevalence of AD (including patients with urticaria). Bakos et al. studying the prevalence of AD in 1997 found 2.53% in boys and 3.18% in girls in children between the age 0–18 (Bakos 1997). Harangi et al. (2003) detected a 15.1% prevalence of AD in school children in Baranya country/Hungary, in 2002 using the Schultz-Larsen questionnaire. The prevalence was slightly higher in cities (16.5%), than in villages (13.7%). Girls showed a slightly higher prevalence of AD than boys: 15.9 and 14%, respectively. The difference between girls and boys was even higher in city-schools (18.2 vs. 14.8%, respectively).

Table III. Correlation between characteristic data of infancy and SCORAD index in patients with AD.

| Data | $r$ | $p$ |
|---|---|---|
| Birth-weight | $-0.0671$ | 0.532 |
| Duration of breast feeding | $-0.2247$ | 0.034* |
| Supplementary feeding | $-0.45$ | 0.670 |
| Begin of cow milk | $-0.0070$ | 0.948 |
| Mixed feeding | $-0.0831$ | 0.439 |

$r$, correlation coefficient; $p$, statistical difference; *, statistically significant difference.

Our survey shows that the prevalence of AD in East-Hungary was 17.5% based on the scores obtained utilizing the Schultz-Larsen questionnaire. Similarly, to previously reported studies (Harangi et al. 2003) we detected a slight female dominance (18.2% girl vs. 16.4% boy), although, the difference was not statistically significant ($p = 0.4143$). Furthermore, no statistically significant difference was found between the prevalence of AD in the big city of Nyiregyháza (17.4%), and the 3 smaller settlements (18.25%). These results indicate that the increase in the prevalence of AD is detectable even in industrially poorly developed regions and thus the disease might be triggered by other factors than industrial pollution alone (Williams 1995).

The high prevalence of AD in East-Hungary was somewhat surprising suggesting that perhaps the provoking factors may comprise the agricultural environment. Nutritional or contact allergic components (like detergents), absence of natural inducers of immune tolerance or antigen-poor environment, all might be taken into account in the factors that contribute to and/or influence AD.

Comparing the prevalence of AD in various age groups of children resulted in a statistically significant dominance of the youngest (0–4 years) over the older (15–19 years) children group ($p = 0.0493$) (Figure 1). The increase in prevalence of AD in the youngest population also reflects the increasing tendency of this disease in Hungary.

Results of dermatological examination proved the validity of the Schultz-Larsen questionnaire. The vast majority of children with a score of $\geq 50$ were found to be suffering from the disease, while those with a score of $< 50$ appeared to be associated with no AD. On the other hand, there was no statistical correlation between the questionnaire's score and the SCORAD index.

It is important to emphasize that our analysis showed a negative correlation between the duration of breast feeding and the SCORAD value ($r = -0.2247$, $p = 0.034$; Table III). Dietary guidance for children with the risk of atopic diseases should strongly recommend the prophylactic breast-feeding and the avoidance of highly allergenic foods within the first year of life. It is believed that prevention of priming the immune system has a beneficial effect in these genetically predisposed children. Trigger factors that have been identified should be avoided, or specific allergen avoidance strategies have to be implemented (e.g. dietary changes, encasing bedding against house dust mite allergen, removal of pets from home, etc.). Prevention of drying of the skin in predisposed patients by creams and emollients is useful to protect against relapsing the disease.

Our results together with the similar survey in Baranya county (Harangi et al. 2003) demonstrate that the prevalence of AD in Hungary is comparable to that recently observed in other surveys in Europe, Japan and United States (Kay et al. 1994, Dotterud et al. 1995, 2001, Sugiura et al. 1998, Williams et al. 1999, Laughter et al. 2000, McNally et al. 2000, Tay et al. 2002). Our results also indicate that the phenomenon of increasing prevalence of atopic diseases detected worldwide is valid in Hungary and therefore appropriate authorities should pay special attention to organize the proper health care of these patients and moreover, prevention of the manifestation of the disease.

### Acknowledgements

We appreciate the cooperation from all parents and children participated in this study and also the support of the Association for Patients with Atopic Dermatitis.

### References

Aberg N, Hesselmar B, Aberg B, Eriksson B. 1995. Increase of asthma, allergic rhinitis and eczema in Swedish schoolchildren between 1979 and 1991. Clin Exp Allergy 25:815–819.

Bakos N. 1997. Prevalence of atopic dermatitis in Hungary European allergy white paper. Brussels: The UCB Institute of Allergy. p 25.

Diepgen TL, Fartasch M, Homstein OP. 1989. Evaluation and relevance of atopic basic and minor features in patients with atopic dermatitis and in the general population. Acta Derm Venereol Suppl (Stockh) 144:50–54.

Dotterud LK, Kvammen B, Lund E, Falk ES. 1995. Prevalence and some clinical aspects of atopic dermatitis in the community of Sor-Varanger. Acta Derm Venereol 75:50–53.

Dotterud LK, Odland JO, Falk ES. 2001. Atopic diseases among schoolchildren in Nikel, Russia, an Arctic area with heavy air pollution. Acta Derm Venereol 81:198–201.

Downs SH, Marks GB, Sporik R, Belosouva EG, Car NG, Peat JK. 2001. Continued increase in the prevalence of asthma and atopy. Arch Dis Child 84:20–23.

Goncz Zs, Szentpeteri J, Mucsi M. 1997. Allergy screening tests in young adolescence European allergy white paper. Brussels: The UCB Institute of Allergy. p 25.

Hanifin JM, Rajka G. 1980. Diagnostic features of atopic dermatitis. Acta Derm Venereol Suppl (Stockh) 92:44–51.

Harangi F, Hartmann A, Lorinczy K, Schneider I, Sebok B. 2003. Prevalence of atopic dermatitis in school children of Baranya county. Orvosi Hetilap 144:429–433.

Iikura Y, Naspitz CK, Mikawa H, Talaricofico S, Baba M, Sole D, Nishima S. 1992. Prevention of asthma by ketotifen in infants with atopic dermatitis. Ann Allergy 68:233–236.

Kay J, Gawkrodger DJ, Mortimer MJ, Jaron AG. 1994. The prevalence of childhood atopic eczema in a general population. J Am Acad Dermatol 30:35–39.

Kunz B, Oranje AP, Labreze L, Stalder JF, Ring J, Taieb A. 1997. Clinical validation and guidelines for the SCORAD index: Consensus report of the European Task Force on atopic dermatitis. Dermatology 195:10–19.

Laughter D, Istvan JA, Tofte SJ, Hanifin JM. 2000. The prevalence of atopic dermatitis in Oregon schoolchildren. J Am Acad Dermatol 43:649–655.

McNally NJ, Williams HC, Phillips DR, Strachan DP. 1958. Is there a geographical variation in eczema prevalence in the UK? Evidence from the 1958 British Birth Cohort tudy. Br J Dermatol 142:712–720.

von Mutius E, Weiland S, Fritzsch C, Duhme H, Keil U. 1998. Increasing prevalence of hay fever and atopy among children in Leipzig, East Germany. Lancet 351:862–866.

Ninan TK, Russell G. 1992. Respiratory symptoms and atopy in Aberdeen schoolchildren: Evidence from two surveys 25 years apart. Br Med J 3048:873–75.

Poysa L, Korppi M, Pietikainen M, Remes K, Juntunen-Bakman K. 1991. Asthma, allergic rhinitis and atopic eczema in Finnish children and adolescents. Allergy 46:161–165.

Schultz Larsen F, Hanifin JM. 1992. Secular change in the occurrence of atopic dermatitis. Acta Derm Venereol Suppl (Stockh) 176:7–12.

Schultz Larsen F, Diepgen T, Svensson A. 1996. The occurence of atopic dermatitis in north Europe: An international questionnaire study. J Am Acad Dermatol 34:760–764.

Severity scoring of atopic dermatitis: The SCORAD index 1993. Consensus report of the European Task Force on atopic dermatitis. Dermatology 186:23–31.

Strom K, Abeck D. 1999. Atopisches Ekzem. In: Traupe H, Hamm H, Hrsg, editors. Pädiatrische Dermatologie. Heidelberg: Springer Verlag Berlin. p 417–433.

Sugiura H, Umemoto N, Deguchi H, Murata Y, Tanaka K, Sawai T, Omoto M, Uchiyama M, Kiriyama T, Uehara M. 1998. Prevalence of childhood and adolescent atopic dermatitis in a Japanese population: Comparison with the disease frequency examined 20 years ago. Acta Derm Venereol 78:293–294.

Svensson A, Edman B, Moller H. 1985. A diagnostic tool for atopic dermatitis based on clinical criteria. Acta Derm Venenereol Suppl (Stockh) 114:33–40.

Tay YK, Kong KH, Khoo L, Goh CL, Giam YC. 2002. The prevalence and descriptive epidemiology of atopic dermatitis in Singapore school children. Br J Dermatol 146:101–106.

Williams H, Robertson C, Stewart A, Ait-Khaled N, Anabwani G, Anderson R, Asher I, Beasley R, Bjorksten B, Burr M, Clayton T, Crane J, Ellwood P, Keil U, Lai C, Mallol J, Martinez F, Mitchell E, Montefort S, Pearce N, Shah J, Sibbald B, Strachan D, von Mutius E, Weiland SK. 1999. Worldwide variations in the prevalence of symptoms of atopic eczema in the International Study of Asthma and Allergies in Childhood. J Allergy Clin Immunol 103:125–138.

Williams H. 1995. Atopic eczema. We should look to the environment. Br Med J 311:1241–1242.

Wuthrich B:. 1994. Atopic dermatitis. Ther Umsch 51:45–54.

Yura A, Shimizu T. 2001. Trends in the prevalence of atopic dermatitis in school children: Longitudinal study in Osaka Prefecture, Japan, from 1985 to 1997. Br J Dermatol 145:966–973.

